# A Perspective on Application of Carbon Quantum Dots in Luminescence Immunoassays

**DOI:** 10.3389/fchem.2020.580033

**Published:** 2020-11-10

**Authors:** Mahdi Hesari, Zhifeng Ding

**Affiliations:** Department of Chemistry, The University of Western Ontario, London, ON, Canada

**Keywords:** carbon quantum dots, luminescence immunoassays, carbon-based nanomaterials, antibody and antigen detection, photoluminescence

## Abstract

Quantum dots (QDs) have been established in our daily life, for instance, in display screens and light-harvesting technologies, mainly owing to their peculiar opto-electronic features. However, toxicity of inorganic QDs, such as CdSe, CdTe, and perovskites, limits their applications in biological environments for medical diagnosis and bio-imaging purposes. A new generation of QDs called carbon quantum dots (CQDs) have been progressing rapidly over the past few years. CQDs have become as popular as other carbon-based nanomaterials such as carbon nanotubes (CNTs), due to their ease of preparation, ultra-small size, biocompatibility, and bright luminescence.

## Introduction

Carbon quantum dots (CQDs) are a newly developed division of carbon-based nanomaterials (e.g., nanodiamond, fullerenes, carbon nanotubes, graphene, and graphene oxide) which are environmentally friendly, chemically stable, and reasonably conductive (Lim et al., 2015; Cayuela et al., 2016; Liu et al., 2016; Namdari et al., 2017; Molaei, 2020). These basic properties have attracted a wide range of researchers to employ CQDs in various applications including biomedical and biotechnological applications (Namdari et al., 2017; Iravani and Varma, 2020), dye-synthesized solar cells (Gao et al., 2020), light-emitting devices, (Yuan et al., 2018; He et al., 2020) imaging and bioimaging (Zhu et al., 2012; Jiang et al., 2015a; Smith and Gambhir, 2017; Panwar et al., 2019; Liu Y. et al., 2020), electrochemical and electrochemiluminescence studies (Wang and Dai, 2015; Fiorani et al., 2019; Wang et al., 2019; Chen et al., 2020; Kour et al., 2020; Zhong et al., 2020), and sensing (Li et al., 2019; Huang et al., 2020; Molaei, 2020; Tajik et al., 2020). More importantly, CQDs possess outstanding optical features, including a bright and tunable luminescence emission within a wide range of wavelengths (Sun et al., 2006; Wang et al., 2017). The emission wavelength is easily altered by the size of CQDs and their composition, which can be obtained using different starting materials and/or synthesis methodology (Jiang et al., 2015a; Lim et al., 2015; Wang et al., 2017; Zhou et al., 2019; Huang et al., 2020; Liu Y. et al., 2020). Thus, this optical tunability of CQDs is being utilized for applications in photodetection platforms (Gao et al., 2016). In this perspective, we summarize the synthesis of CQDs and explain the influences of doping elements on the opto-electronics of these nanomaterials, while the main focus remains on their photochemistry. We further review the surface functional modification of CQDs, which enables researchers to exclusively couple them with a variety of antibodies, antigens, and proteins. The fundamental applications of CQDs in luminescent immunoassay detections of a broad range of biological samples are highlighted. In this perspective, we cover the most impactful applications of CQDs in immunoassays from 2014 to date.

## Synthesis of Carbon Quantum Dots

The ease of preparation, characterization, and storage of CQDs have made these nanomaterials available and accessible for different proposes (Namdari et al., 2017; Iravani and Varma, 2020). Here, we summarize various synthesis methods, e.g., top-down and bottom-up methods, which are implemented to make CQDs, where the main difference between the two, are the availability and preference of the starting materials.

### Top-Down Method

In this method a larger carbon source material has been used. For example, in an electrochemical oxidation (also known as exfoliation) approach a constant potential (e.g., 5 V) is applied to a graphite rod immersed in an electrolytic solution (Fu et al., 2019). This technique, in fact, results in decomposition of a graphitic texture and the formation of smaller graphitic particles with highly oxidized surface functionalities. Our group has utilized multiwalled carbon nanotubes to generate graphene quantum dots via electro-exfoliation in a potentiodynamic process (Zhou et al., 2007). Alternatively, electrolysis of a carbon source, e.g., coke, using a two-electrode system including Pt and steel plates under constant current conditions, generates CQDs (He M. et al., 2018). Chemical oxidation of large organic structures e.g., polyamide systems and carbohydrates, in an acidic condition using a strong acid ultimately produces CQDs. For example chemical oxidation of tris(4-aminophenyl)amine (TAPA) in the presence of tert-butyl hydroperoxide (TBHP) and hydrochloric acid results in the formation of CQDs (Liu Y. et al., 2020). Furthermore, one can use high energy external resources such as ultrasonic (Dang et al., 2016), micro-plasma (Ma et al., 2019), or arc discharges (Arora and Sharma, 2014) to break down large organic molecules or other carbon sources into smaller pieces.

### Bottom-Up Method

**Scheme 1:**
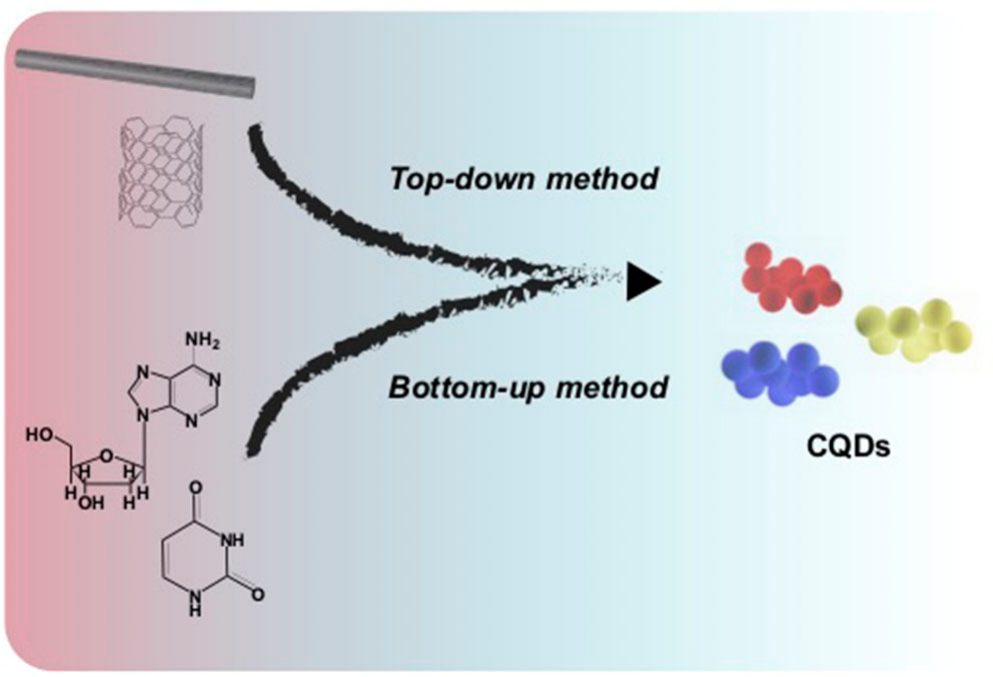
An illustration of preparation methods for carbon quantum dots (CQDs). Different colors of the CQDs can be obtained depending on the precursors and synthetic strategies.

This method works by fusing and reconstructing small organic molecules to synthesize particles. There are different protocols method) (e.g., microwave assisted, pyrolytic process, and template to synthesize CQDs following the bottom-up approach (Tajik et al., 2020); however the hydrothermal method is the most popular process owing to its easy setup and the broadness of useable reactive materials (Shen and Xia, 2014; Jiang et al., 2015b; Zhao et al., 2015; Wu et al., 2017; Mondal and Saha, 2019; Huang et al., 2020; Liu Y. et al., 2020). Indeed, in this approach small organic molecules, such as aspartic acid (Mondal and Saha, 2019), and cysteine (Huang et al., 2020), or easily decomposable organic biomasses such as garlic (Zhao et al., 2015), or grapefruit peel (Xiao et al., 2018) are used as starting materials. Practically, organic substances are dissolved in a solvent and transferred into a Teflon-lined autoclave and heated to a specific temperature to complete the reaction. Scheme 1 depicts a summary of CQD synthesis methods.

In all aforementioned methods, the final mixture, including residual starting materials and CQD particles with different sizes, are transferred into a dialysis bag or loaded onto a chromatographic column for further purification (Liu et al., 2018). Several techniques such as FT-IR, UV-visible, and photoluminescence (PL), x-ray photoelectron spectroscopy (XPS), and transmission electron microscopy (TEM) have been extensively employed to characterize CQDs (Liu et al., 2016; Fu et al., 2019; Ma et al., 2019; Liu Y. et al., 2020). For instance, FT-IR and XPS have enabled researchers to identify and/or confirm surface functionality groups (e.g., –OH, –NH-, –SH), UV-visible spectroscopy along with PL have been used to investigate optical features of CQDs (vide infra). Furthermore, high-resolution TEM (HRTEM) helps to not only identify the size of individual particles, but it is also possible to recognize lattice fringes with an interplanar spacing.

## Photophysics of Carbon Quantum Dots

**Figure 1:**
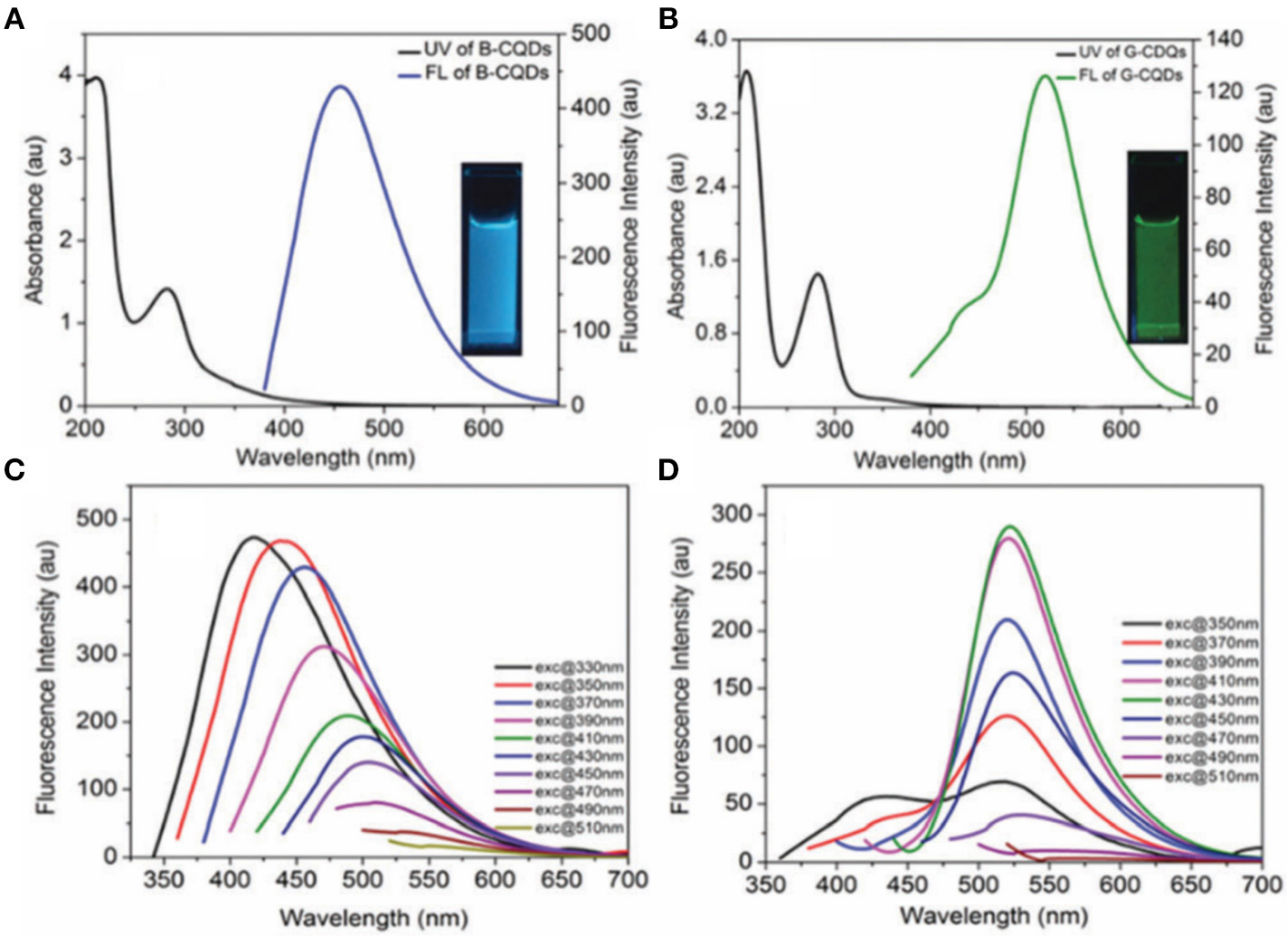
Absorption and photoluminescence spectra of **(A)** blue and **(B)** green emissive CQDs. **(C)** and **(D)** represent the excitation-dependence spectroscopy of the blue and green CQDs at different excitation wavelengths. Panels reproduced from Singh et al. (2018). Copyright 2018, Royal Society of Chemistry.

Photoluminescence is a common and important property of CQDs, which empowers many researchers to make use of CQDs for various purposes such as light-emitting device fabrications (Yuan et al., 2018; He et al., 2020), and more widely for detecting diverse target species e.g., metal ion detection (Gao et al., 2016; Liu et al., 2017). The overall photophysical responses of CQDs in the course of light absorption and emission processes are based on an isolated network of π-bonding originating from sp^2^ carbon backbones. The critical point is that if the π-bonds form an extended network, similar to what is observed in carbon nanotubes (CNTs), graphite, and graphene, there is no or negligible light interaction. This may be due to the continuous non-radiative recombination among photogenerated electrons and holes (Lim et al., 2015). In fact, the light absorption in CQDs occurs almost in the wide range of the wavelength, however the dominant emission at a specific wavelength is governed by the population of a peculiar domain, and the inherent surface functionality. The excitation-dependent emission behavior is one of the typical photophysical characteristics of CQDs that originates from the surface state of these nanomaterials. For instance, Goel and co-workers prepared two fluorescent CQDs with blue and green emissions using hydrothermal and chemical oxidation methodologies, respectively (Singh et al., 2018). The UV-visible and PL spectra resulting from the blue emissive (B-CQD) and green emissive (G-CQD) materials are shown in Figures 1A,B with an absorption peak at ~280 nm and PL emissions at 450 and 510 nm, respectively. Interestingly, the B-CQDs revealed an obvious excitation wavelength dependency at a 20-nm wavelength increment (Figure 1C), while G-CQDs show basically no dependency (Figure 1D). The excitation dependency property of CQDs is an extraordinary feature that leads to tunable photoluminescence nanomaterials. It is worth mentioning that the quantum efficiency and tunablity of PL are improved by using a passivation agent such as poly(propionyl ethyleneimine-co-ethyleneimine) (PPEI-EI) (Sun et al., 2006), or polyethylene (PE) (Fernandes et al., 2020). In fact, the polymeric layer(s) could extend emission life time, and enhance the PL quantum yield, that depends on π-π^*^ and n–π^*^ transitions across the carbon backbone and also surface functionalities (Dimos, 2018).

### Effect of Doping and Surface Alteration on Photophysical Features

The replacement of the main carbon backbone by a soft element e.g., S (Yang et al., 2016; Yao et al., 2019), Se (Yang et al., 2014), N (Jiang et al., 2015b; Chen et al., 2016; Carolan et al., 2017; He S. et al., 2018; Yao et al., 2019), P (Venkateswara Raju et al., 2020), B (Wei et al., 2020), or both N and S (Zhang et al., 2018), and a hard element e.g., Fe (Zhuo S. et al., 2019) and Ag (Zhuo S. J. et al., 2019) results in the formation of a doped CQD. For example, Raju et al., autoclaved tri-sodium citrate in the presence of phosphoric acid at 180°C for 12 h resulting in the formation of a p–doped CQD, which was further used to detect Cu^2+^ ions (Venkateswara Raju et al., 2020). A yellow emission B and N CQD was also synthesized by mixing o-phenylenediamine with boric acid in an autoclave apparatus. The resulting co-doped CQD revealed a ~14% PL quantum yield (Wei et al., 2020). Liu et al. produced a triply-doped CQD consisting of B, N, and S in a one-pot hydrothermal reaction with an emission at ~600 nm (Liu et al., 2017). It has also been shown that the more electronegative the doping element, the more red-shift occurs in the luminescence emission (Yang et al., 2014). Overall, doping approaches contribute significantly to the improvement of the PL quantum efficiency and provide alternations of the emission wavelengths, by creating the population of an electron rich domain and sometimes more defects. Xu et al. explored the effect of edge functionality on the luminescent emissions of CQDs. They altered the inherent functional groups such as –COOH and –C=O to phosphate, sulfite, and amine by treating CQDs in the presence of phosphoric acid, sulfuric acid, and nitric acid, respectively. They observed that when the –COOH and –C=O groups were presence at the edge of CQDs, PL quantum efficiency reached to ~9%, while introducing the –${\text{PO}}_{4}^{2-}$, –${\text{SO}}_{3}^{-}$, and –NH_2_ groups improved the PL quantum efficiency to 10.3, 29.7, and 18.7%, respectively.

## Application of Carbon Quantum Dots in Immunoassays

Ease of preparation, chemical and biological compatibility, well-defined luminescence signal with tunable wavelength options, diverse surface functionality for further modification, low electrical resistance, and acceptable conductivity, all make CQDs an exceptional candidate for detection technology. There are several designed detecting systems that are constructed around CQD luminescence signals measuring a diverse range of analytes in biological environments, for instance, aptasensor for ochratoxin A detection by Bi et al. (2020). In this section, we focus on recent developments on using CQDs for immunoassays. In fact, CQDs have been mainly utilized in detection platforms owing to their stable luminescence emission (Xiao et al., 2018; Dong et al., 2019a; Yao et al., 2019; Alarfaj et al., 2020; Liu G. et al., 2020). In the past decade, luminescence-based immunoassays have become powerful tools in the field of biological and clinical diagnoses, and microbial investigations. These nanomaterials have been fabricated in point-of-care (POC) systems along with antigen–antibody binding interactions and enzyme–substrate reactions. Their optical properties have been altered and applied through different approaches in which the optical signal was quenched, recovered, or enhanced for the purpose of detection.

**Figure 2:**
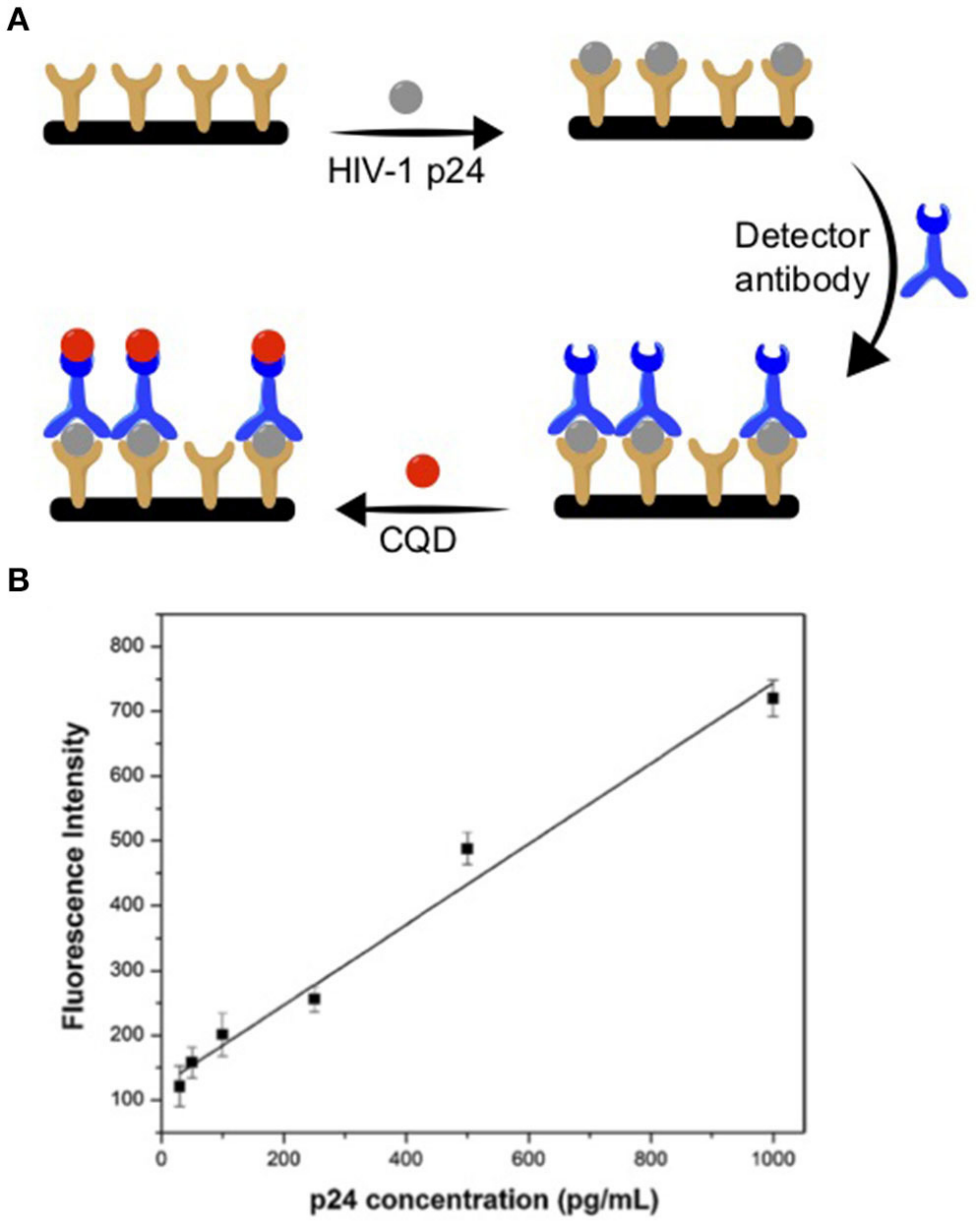
**(A)** HIV-1 p24 antibody-antigen detection device fabrication using CQD. **(B)** Exemplar of p24 antigen detection curve. Panels reproduced from Chunduri et al. (2016). Copyright 2016, Springer.

For example, Chunduri et al. designed an early stage detection of HIV through a POC system (Chunduri et al., 2016). They synthesized nitrogen doped (N-doped) CQDs using citric acid and ethylenediamine as carbon and nitrogen precursors via a one-pot hydrothermal reaction. These CQDs were decorated with streptavidin (CQD-SA) and have been used as fluorescent labels to measure the HIV-1 p24 antigen on both microwell plates and microfluidic chips. The microfluidic devices were machined on a 100 × 100 × 1.5 mm acrylic plaque that was used as a template for polydimethylsiloxane (PDMS) to replicate the microchip pattern for further use. The next step for the device was loading the captured antibody into micro channels with a micropipette and incubating it. Next, the HIV-1 p24 antigen sample solutions were loaded. The loosely bound bonded HIV-1 p24 was then washed away with a buffer. Later, the detector antibody was added to each channel to assemble the antibody–antigen–antibody sandwich immunocomplex. Finally, the CQD-SA solution was loaded into each channel and the streptavidin–biotin coupling reaction was allowed to complete. Figure 2A shows a summary of the fabrication process. The fluorescent signals from the sandwich immunocomplex were then recorded as an analytical signal using a spectraMax M5 fluorescence reader. Figure 2B shows an exemplary p24 measurement using the device based on a CQD luminescence signal in a pg/mL detection range with an *R*^2^ = 0.9737 linear correlation. The inexpensive preparation of CQD and the stable luminescence signal made this device cheaper and more efficient than conventional ELISA immunoassays. The advantage of this type of fabrication is its ease of assembly with minimal required steps.

**Figure 3:**
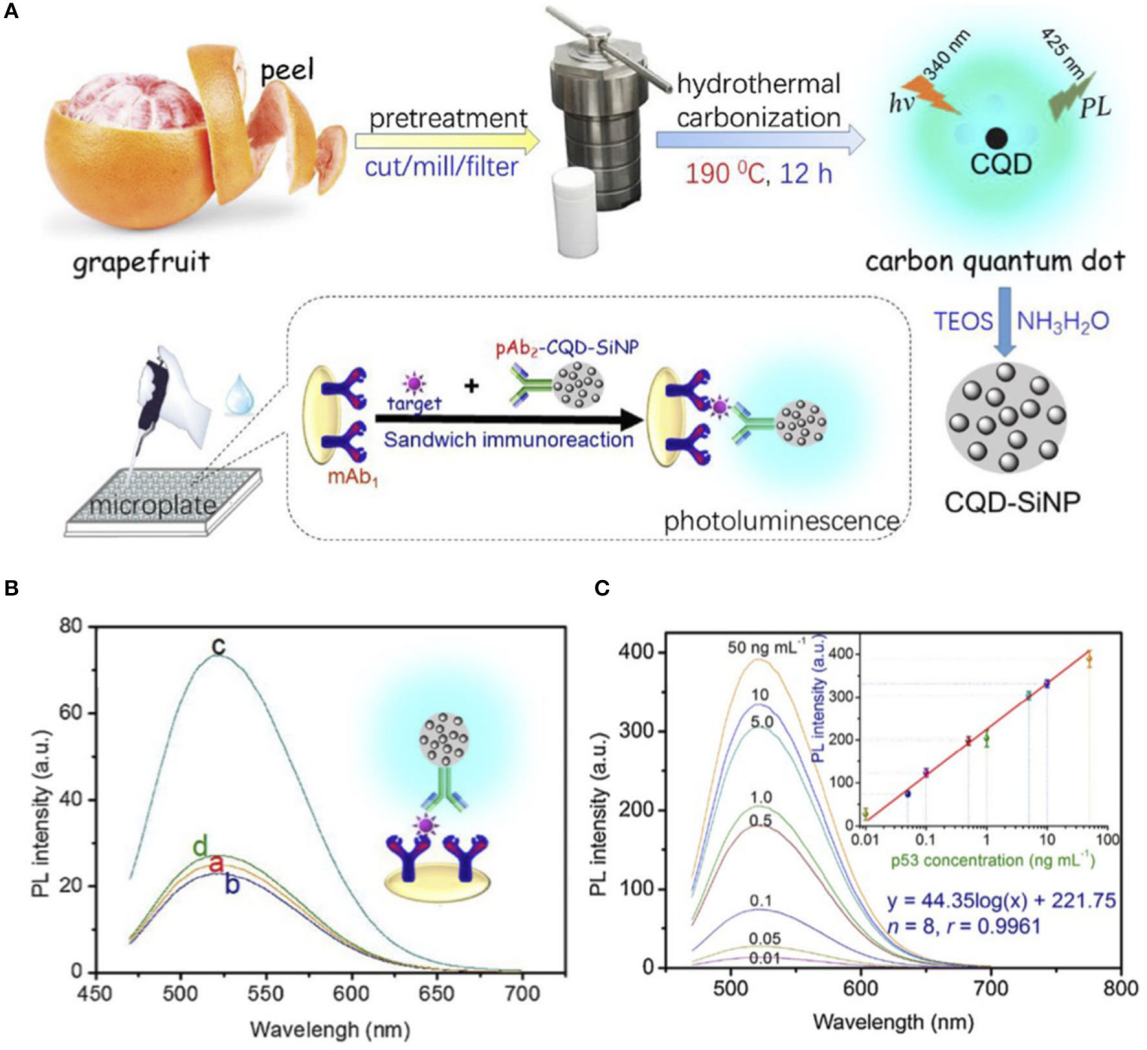
**(A)** Preparation, encapsulation, and assembly steps of CQDs used for p53 protein luminescence detection. **(B)** PL emission spectra of (a) mAb_1_-modified microplate, (b) mAb_1_- modified microplate with 0.05 ng/mL p53, (c) mAb_1_- modified microplate with 0.05 ng/mL p53 and pAb_2_-CQD-SiNP, and (d) mAb_1_- modified microplate with pAb_2_-CQD-SiNP. **(C)** Luminescence emission spectra of the designed immunoassay for the various p53 standards along with the corresponding calibration curve. Panels reproduced from Xiao et al. (2018). Copyright 2018, Elsevier.

Xiao et al. prepared water-soluble CQDs using grapefruit peel treated under hydrothermal conditions at 190°C for 12 h (Xiao et al., 2018). The resulting CQDs with an average size of 4.2 ± 0.1 nm emit light at 425 nm after excitation at 340 nm. Figure 3A represents the summary of the preparation steps. In order to boost the luminescence signal, the CQDs were mixed with a silica precursor. The resulting CQDs-silica nanoparticle (CQD-SiNP), with an average size of ~16 nm, was functionalized with the polyclonal anti-p53 antibody (CQD-SiNP-pAb_2_). The luminescence wavelength of the CQD-SiNP was red shifted owing to the inner-filter effect. To fabricate the detection system, a microplate was decorated with the monoclonal mouse anti-p53 antibody (mAb_1_). To join CQD-SiNP-pAb2 and the mAb1 the target protein, p53 was added into the microplate. They found that a strong signal could be produced by adding 0.05 ng mL^−1^ p53 and pAb_2_-CQD-SiNP into a mAb_1_- modified microplate (Figure 3B). There is a linear relationship (0.01–50 ng/mL) between the detected signal and the added known concentration of the target protein, p53 (Figure 3C). They further found that this method with a limit of detection (LOD) of 2.7 pg/mL works as good as a commercial ELISA kit with a LOD of 65 pg/mL, while it is less expensive to fabricate. Indeed, the luminescence signal improvement facilitated the detection sensitivity and responsivity.

In another strategy, luminescence emission quenching of CQDs driven by antibody interaction was also employed in immunoassay measurements. Yao et al. synthesized S- and N-doped CQDs (S/N-CQDs) with luminescence emissions at 405 nm upon excitation at 350 nm (Yao et al., 2019). The luminescence enhancement of the S/N-doped CQDs was investigated. They proposed a potential mechanism for luminescence improvement for S/N-doped CQDs that was based on a higher population of excited electrons of the doped CQD relative to non-doped ones along with a red-shift emission. They found that if the S/N-doped CQDs coupled with an antibody, e.g., clenbuterol (Clen) antibody (Ab) through amine–amine coupling, the original luminescence intensity of the S/N-doped CQDs would be quenched. Thus, one can use this luminescence signal change as an analytical detection signal to measure Clen in biological environments. In fact, attaching and de-attaching the CQD to the antibody created the luminescence signal difference, ΔF. To establish the signal alternation in the presence and absence of Clen, various concentrations of Clen were added to a solution of S-N-CQD solution and the corresponding luminescence spectra were recorded. The extracted ΔF showed a linear relationship vs. the concentration of Clen, that is an indication of the strong potential application of the strategy to detect Clen in various samples.

**Figure 4:**
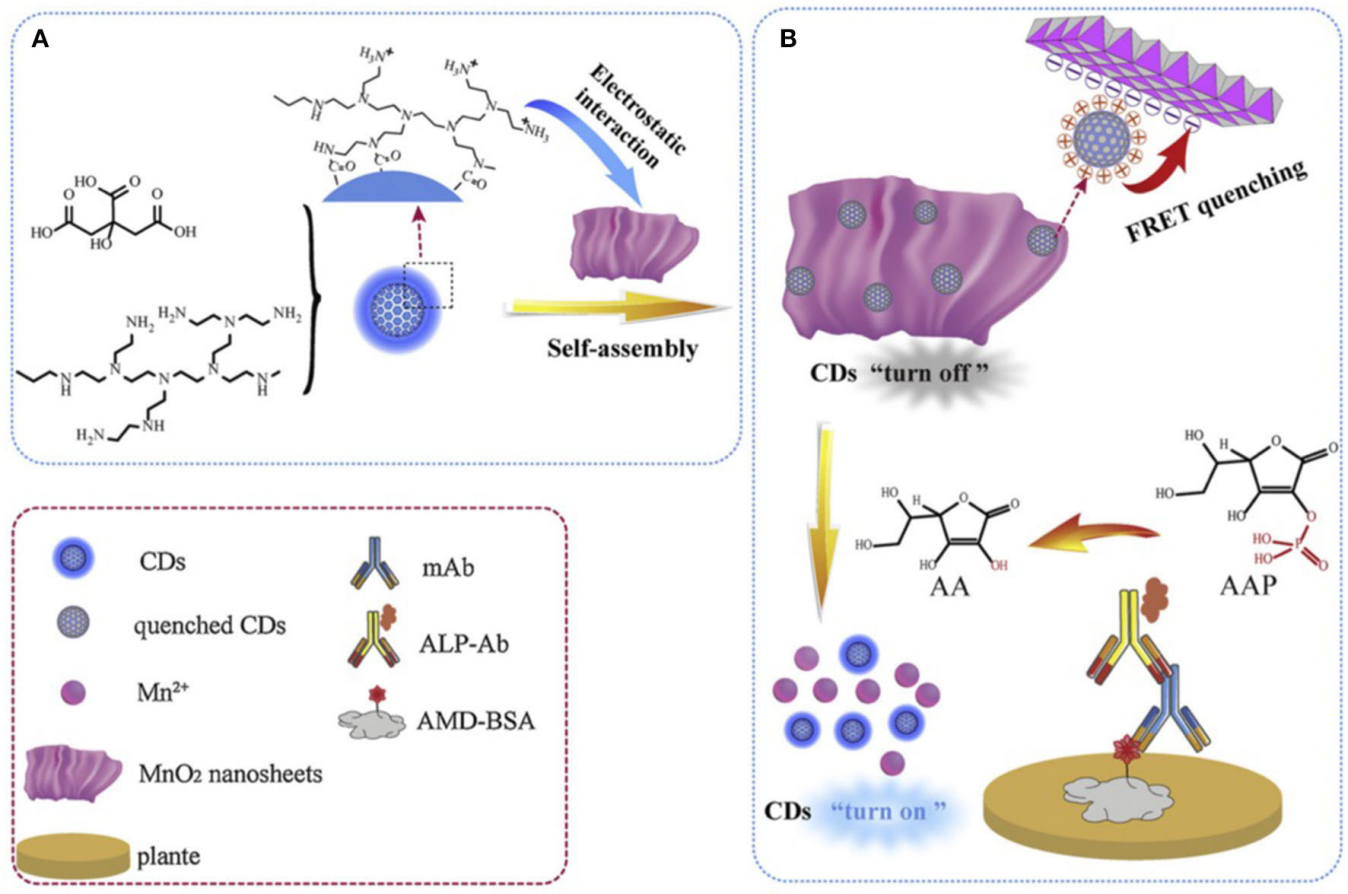
**(A)** Synthesis and **(B)** fabrication of designed luminescence immunoassay to detect amantadine (AMD). Panels reproduced from Dong et al. (2019b). Copyright 2019, Elsevier.

The sensitivity and selectivity of immunoassay measurements based on CQD luminescence signals, and the importance of residual harmful chemicals detection in food chemistry have encouraged Shen and co-workers to expand CQD application in the food industry (Dong et al., 2019b). In their study, the luminescence signal recovery was employed as a detection signal for amantadine (AMD). The surface functionalized CQD was prepared by mixing citric acid and polyethyleneimine branched (PEI, MW = 10,000) in a Teflon-lined autoclave, which was heated at 180°C for 6 h. The resulting CQDs with charged polymeric amine-based coating (p-CQDs) emits at ~ 450 nm with a quantum yield of 41.7% and an average size of 8 nm. Owing to the presence of ammonium moieties on the surface of CQDs they interacted strongly with the negatively charged MnO_2_ nanosheets layer (Figure 4A). The resulting nanocomposite (i.e., p-CDs@MnO_2)_ quenched the original luminescence of CQDs via Forster resonance energy transfer (FRET) between CQDs and MnO_2_ NSs. A further luminescence-based immunoassay was fabricated by placing an antigen, AMD-BSA (Wang et al., 2018), on a microplate. In fact the AMD-BSA acted as an anchor to build the rest of the assay (Figure 4B).

Next an antibody, anti-AMD mAbs, was added to react with the absorbed BSA-AMD antigen. Then an ALP-labeled secondary antibody was introduced. This strategy could result in a lower amount of the required ALP-labeled secondary antibody as a reactive catalysis site. In fact, the ALP-labeled secondary antibody acted as a catalyst for the hydrolysis of 2-phosphoascorbic acid trisodium salt (AAP) to ascorbic acid (AA). At this stage AA triggered p-CDs@MnO_2_ dissociation, that recovered the luminescence of the CQDs which served as the detection signal. Indeed, this is a sophisticated approach for the detection and measurement of an important food poisoning chemical. They also found that the designed system was more sensitive to AA rather than AAS. Further they assessed the selectivity of the immunoassay in the presence of a series of non-targeted chemicals such as ribavirin, moroxydine, acyclovir, acetaminophen, chloramphenicol, enrofloxacin, and sulfamethazine. This system responded to 0.048–1.1 ng mL^−1^ of AND with a LOD of 0.035 ng mL^−1^.

**Scheme 2:**
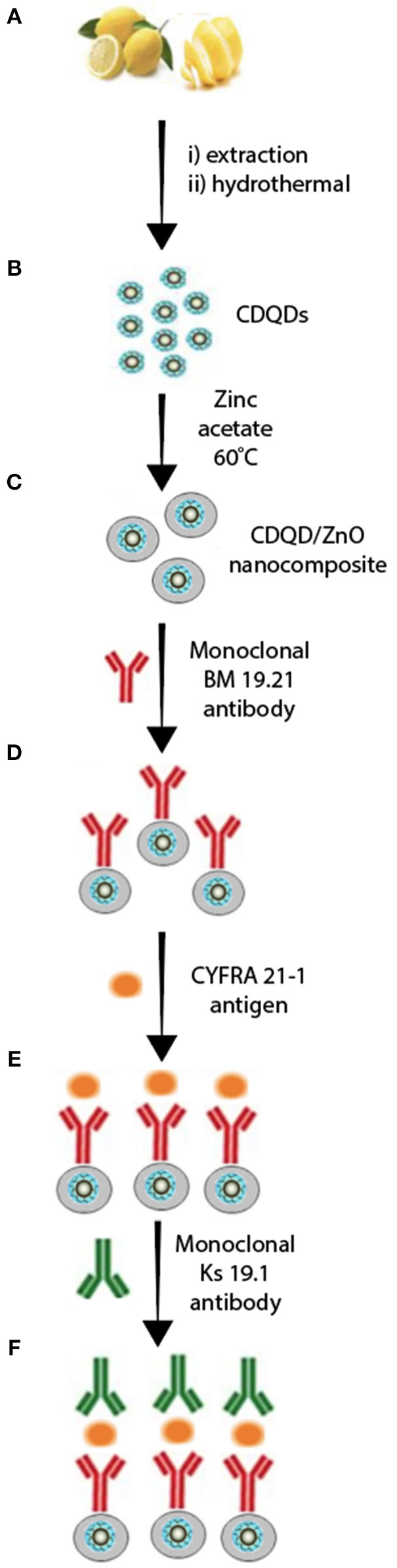
Luminescence immunoassay based on CQDs/ZnO nanocomposite and antibody-antigen-antibody system. **(A,B)** CDQD synthesis, **(C)** ZnO coated CDQD, **(D)** decorated CDQD/ZnO with 8M 19.21 antibody, **(E)** fabricated CDQD/ZnO/antibody with CYFRA 21.1 antigen, **(F)** final assembly of CDQD/ZnO/antibody/antigen/antibody assay. Panel reproduced from Alarfaj et al. (2020). Copyright 2020, Springer.

Recently, Oraby and co-workers reported a nanocomposite consisting of CQD (size ~ 5 nm, λ_abs._= 224 nm, and 280 nm, λ_em_= 452 nm) decorated with zinc oxide (i.e., CQD/ZnO, λ_abs._= 370 nm, λ_em_= 520 nm) (Alarfaj et al., 2020) (Schemes 2A,B). The resulting highly stable CQD/ZnO composite (Scheme 2) was further decorated with a non-conjugated monoclonal BM 19.21 antibody via the reaction of the surface carboxylic groups with amine sites of the peptide amide of the antibody backbone in the presence of N-hydroxysuccinimide (NHS) and carbodiimide hydrochloride (EDC) (Scheme 2D). The CQDs/ZnO-BM 19.21 luminescence immunoassay was further constructed and utilized to detect lung cancer at early stages using the antigen cytokeratin-19 fragment (CYFRA 21-1) as a tumor marker (Scheme 2E). The antibody-antigen-antibody immunoassay sandwich was completed by introducing the monoclonal Ks 19.1 antibody (Scheme 2F).

It was found that CQDs/ZnO-BM 19.21 immunoassay systems work within a wide range of linear detection (i.e., 0.01–100 ng. mL^−1^) with a LOD of 0.008 ng mL^−1^ which is easier to use than other techniques such as enzyme and electrochemiluminescence (ECL) immunoassays. For instance, in order to utilize an electrochemical or ECL immunoassay sensor, the modified CQDs need to be assembled on the surface of an electrode. This, occasionally, results in reduced stability in the immunosensor. Further specific tailoring of the CQDs functionalities is needed (Chen et al., 2020; Zhong et al., 2020).

## Perspective and Future Work

Immunoassay detection is a well-established technique to measure various target molecules in many biological matrices. There are a vast number of approaches to design a selective and sensitive immunoassay system, while the unique inherent prosperities of CQDs have assisted researchers to achieve discriminatory systems with much higher precision when detecting crucial analytes. For example, as we summarized in the current article, CQD luminescence emission provides a well-defined detecting signal that is sensitive to reaction and interaction in the analyte solution. It is important to highlight that the variety of original chemical functionality such as –OH, –COOH, –C=O, and upon doping, –NH_2_, –${\text{SO}}_{3}^{-}$, –${\text{PO}}_{4}^{2-}$ provide straightforward and easy access platforms that outperform many other options. For instance, upon the appearance of COVID-19 as a worldwide pandemic, one can use CQDs along with an antibody-antigen-antibody strategy to detect COVID-19 in various media (Nabel and Shiver, 2019; Pinto et al., 2020; Shi et al., 2020). Here we highlighted the most recent advanced procedures on the luminescence features of CQDs. Alternatively, based on easy modifications with antigens and antibodies and good conductivity of CQDs one can develop electrochemical and electrochemiluminescence (ECL) studies toward various detecting proposes including immunoassay sensing (Zhou et al., 2014; Chen et al., 2020). This approach can be further expanded in solid-state electrochemical and ECL investigations by employing surface functionalities of CQDs and generate background free signals toward detection.

## Data Availability Statement

The original contributions presented in the study are included in the article/supplementary material, further inquiries can be directed to the corresponding author/s.

## Author Contributions

MH and ZD defined the topic and wrote the manuscript.

## Conflict of Interest

The authors declare that the research was conducted in the absence of any commercial or financial relationships that could be construed as a potential conflict of interest.
